# Disentangling the potential roles of the human gut mycobiome and metabolites in asthma

**DOI:** 10.1002/ctm2.1012

**Published:** 2022-08-28

**Authors:** Chunrong Huang, Wei Tang, Ranran Dai, Ping Wang, Guochao Shi, Wei Du, Yingmeng Ni

**Affiliations:** ^1^ Department of Pulmonary and Critical Care Medicine, Ruijin Hospital Shanghai Jiao Tong University School of Medicine Shanghai People's Republic of China; ^2^ Institute of Respiratory Diseases, Ruijin Hospital Shanghai Jiao Tong University School of Medicine Shanghai People's Republic of China; ^3^ Shanghai Key Laboratory of Emergency Prevention, Diagnosis and Treatment of Respiratory Infectious Diseases, Ruijin Hospital Shanghai Jiao Tong University School of Medicine Shanghai People's Republic of China

Dear Editor,

Although previous studies reported associations between gut bacterial dysbiosis and asthma,^1^, ^2^ few addressed the gut fungal microbiota (mycobiome) and metabolome in adult asthma patients. Here, we firstly explored the gut mycobiome of 21 healthy controls (HCs) and 38 asthma patients, with 12 receiving no ICS treatment (NT) and 26 receiving inhaled corticosteroids (ICS; Table S1, Supporting information include detailed methodology). All participants were omnivores. Nine and 16 patients in NT and ICS groups, respectively, experienced allergic rhinitis. Patients on ICS exhibited longer disease duration than those on NT. Compared with HC, patients prescribed NT and ICS showed worse lung function (decreased predicted percentage of forced expiratory volume in 1 s (FEV1%pre)). No significant differences in species coverage were observed among the groups. Compared with HC, patients on ICS showed significantly lower fungal richness (observed species), evenness (Pielou_e), and diversity (Shannon) (Fig. 1A). This is inconsistent with previously reported bacteriome results inplying indiscriminate bacterial richness or diversity in adult asthma patients.^3^, ^4^ These data suggest a different influence of asthma state on gut mycobiome and bacteriome; however, validation via large‐scale studies is needed. Both the observed species and Shannon indices negatively correlated with disease duration and positively correlated with sputum neutrophil percentages (Fig. 1B). Principal coordinate analysis (PCoA) using Bray–Curtis distance observed significant clustering among all groups, indicating differing community composition (Adonis test, *p* = .026; Fig. 1C; Table S2).

**FIGURE 1:**
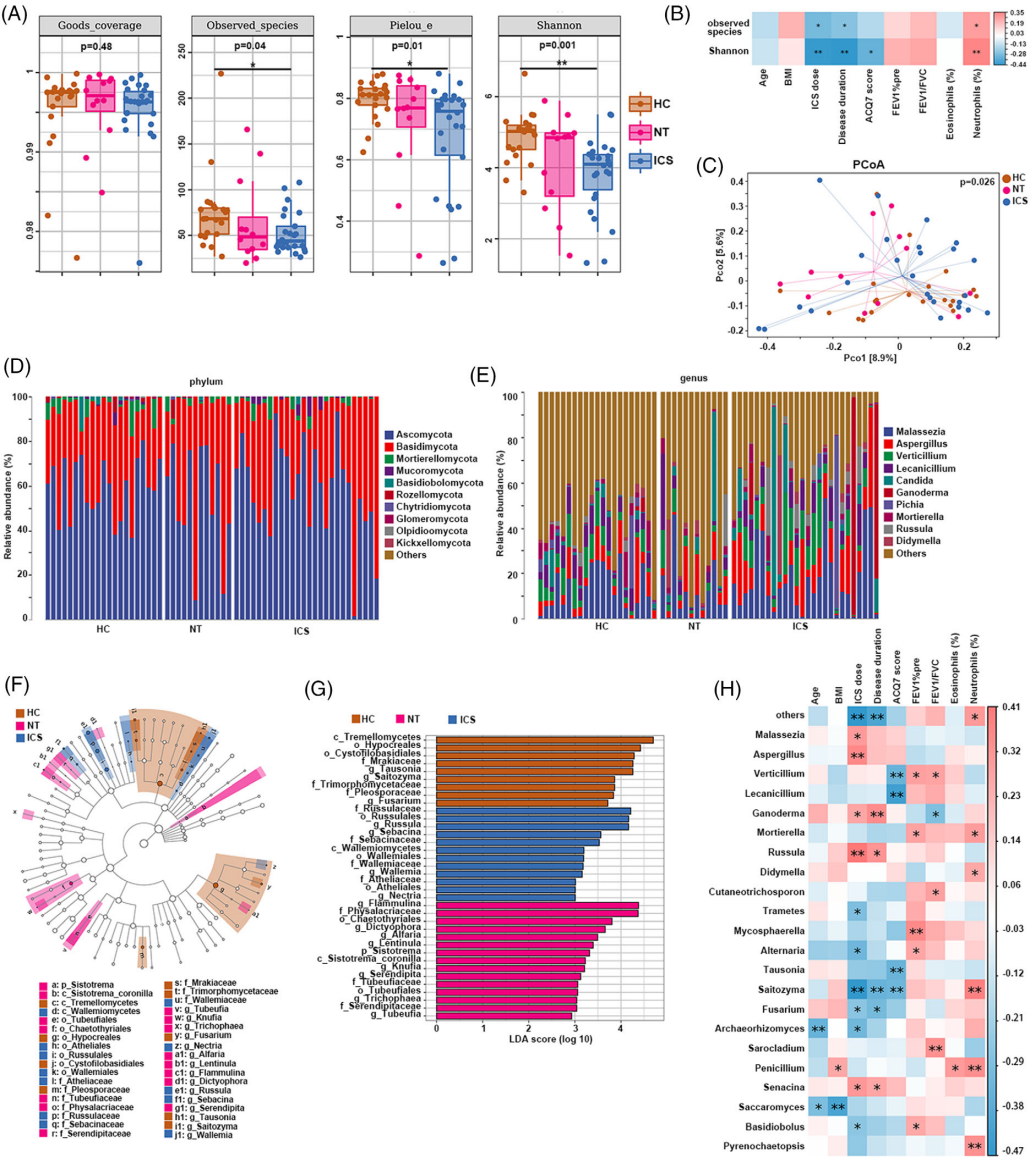
Estimations of gut mycobiome among healthy control (HC), NT, and inhaled corticosteroid (ICS) groups. (A) Comparison of α diversity including species coverage, richness (observed species), evenness (Pielou_e), diversity (Shannon) metrics among HC, NT, and ICS groups. Kruskal–Wallis test with Dunn's post‐test. *p < .05. **p < .01. (B) Heatmap showing associations between mycobiome α diversity indices and clinical characteristics of asthma using Spearman correlation. *p < .01. **p < .001. Red indicates positive correlation; blue indicates negative correlation. BMI: body mass index; ACQ7 score: Asthma Control Questionnaires; FEV1%pre: forced expiratory volume in 1 s percentage predicted; FEV1/FVC: forced expiratory volume in 1 s/forced vital capacity percentage; eosinophils (%): the percentages of sputum eosinophils; neutrophils (%): the percentages of sputum neutrophils. (C) Principal coordinate analysis (PCoA) of Bray–Curtis distance matrix analysis. (D,E) Relative abundance of dominant enteric fungi at the phylum and genus level. (F) Taxonomic cladogram obtained from linear discriminant analysis effect size (LEfSe) analysis showing differentially abundant taxonomic clades. (G) Histogram of the linear discriminant analysis (LDA) scores computed for taxon differentially abundant among the HC, NT, and ICS groups. Only taxa meeting an LDA significant threshold > 2 are shown. (H) Associations between fecal mycobiome and clinical characteristics of asthma using Spearman correlation. *p < .01. **p < .001. Only taxa significantly correlated with any of clinical indices were showed. Red indicates positive correlation; blue indicates negative correlation. BMI: body mass index; ACQ7 score: Asthma Control Questionnaires; FEV1%pre: forced expiratory volume in 1 s percentage predicted; FEV1/FVC: forced expiratory volume in 1 s/forced vital capacity percentage; eosinophils (%): the percentages of sputum eosinophils; neutrophils (%): the percentages of sputum neutrophils

Figures 1D,E, and S1 showed the gut mycobiome composition at phylum, class, family, and genus levels. The dominant phyla were Ascomycota, Basidiomycota, and Mortierellomycota; the dominant genera were *Malassezia* and *Aspergillus*. Linear discriminant analysis effect size was used to investigate mycobiome biomarkers (discriminately enriched fungi) at various taxonomic ranks. This revealed that one class, three families, and three genera, including *Fusarium*, were discriminately enriched in HC. One phylum, one class, three families, and eight genera (*Dictyophora*, etc.) were enriched in the NT group, the polysaccharides of *Dictyophora* was demonstrated to have several physicochemical and biological properties.^5^ In the ICS group, one class, four families, and four genera were enriched (Fig. 1F,G), among which *Russula* is a valuable, medicinal fungus with anti‐inflammatory bioactivities.^6^ The correlations between the mycobiome and clinical indices showed that disease duration correlated positively with the abundance of *Ganoderma*, *Russula*, and *Senacina* and negatively with the abundance of *Saitozyma* and *Fusarium*. Sputum neutrophils (%) were positively associated with the abundance of *Mortierella*, *Didymella*, *Saitozyma*, *Penicillium*, and *Pyrenochaetopsis* (Fig. 1H).

Comparing the gut mycobiome in asthma patients with different disease severities against HC revealed that severe patients exhibited significantly lower fungal richness (observed species), evenness (Pielou_e), and diversity (Shannon), and mild‐to‐moderate patients showed significantly lower fungal diversity (Shannon) (Fig. S2A–2D). PCoA indicated different community compositions among HC and patients with mild‐to‐moderate and severe disease (Adonis test *p* = .008; Fig. S2E; Table S2).

Metabolites synthesized in the gut possess bioactivities against diseases.^7^ To investigate the gut metabolome in asthma, 24 HC, and 23 and 31 patients in NT and ICS groups, respectively, were enrolled. Patients receiving ICS exhibited longer disease duration than those in NT group; patient in NT and ICS groups showed worse lung function (lower FEV1%pre and FEV1/forced vital capacity) than HC (Table S3). Orthogonal partial least‐squares discriminant (OPLS‐DA) analysis showed dots in dark blue clearly separated from green dots (NT vs. HC, and NT vs. ICS) in electrospray ionization positive (ESI+) and negative (ESI–) modes, indicating different metabolic profiles between HC and NT, or between NT and ICS groups (Fig. 2A,B). With variable importance in projection >1 and *p* < .05, nine (ESI+) and three (ESI–) discriminate metabolites were found between NT and HC groups (Fig. 2C, Table S4). For example, sphingosine levels were upregulated in the NT group. Sphingosine/sphingosine‐1‐phosphate was considered to trigger asthma.^8^ NT group also possessed decreased anti‐inflammatory metabolite (e.g., vitamin A and L‐fucose) levels.^9^, ^10^ Six (ESI−) and three (ESI+) differential metabolites were found between NT and ICS group (Fig. 2D, Table S5). Strong correlations were found between these metabolites (Fig. 2E,F). Additionally, Kyoto Encyclopedia of Genes and Genomes enrichment analysis of differential metabolites revealed apoptosis to be most affected in the NT group, followed by necroptosis (Fig. 2G); glycine, serine, and threonine metabolism were significantly affected in patients receiving ICS (Fig. 2H).

**FIGURE 2:**
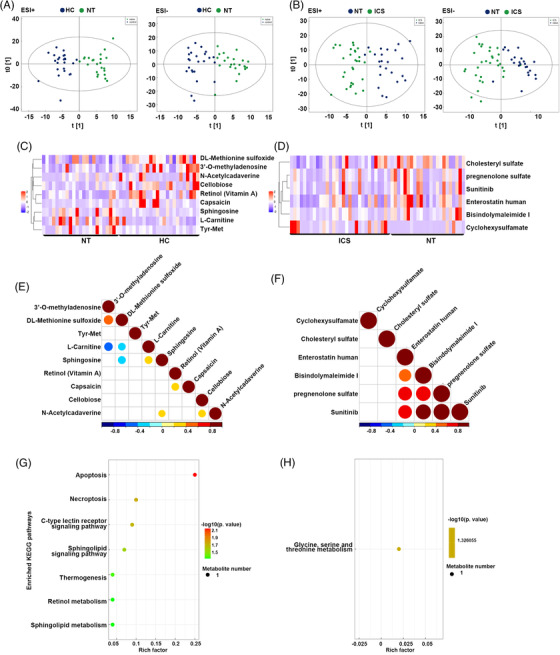
Differential metabolites, correlation analysis, and enrichment analysis in fecal samples. (A) The orthogonal partial least‐squares discriminant analysis (OPLS‐DA) score plots between healthy control (HC) and NT groups in electrospray ionization positive mode (ESI+) and negative mode (ESI–). OPLS‐DA was used to reveal metabolic differentiation between groups. Cluster of dots with the same colour represents a similar metabolic profile, a clear separation between dots with different colours represents the difference of metabolic profiles between groups. Dark blue: HC group, green: NT group. (B) The OPLS‐DA score plots between NT and inhaled corticosteroid (ICS) groups in ESI+ mode and ESI– mode. Cluster of dots with the same color represents a similar metabolic profile, a clear separation between dots with different colours represents the difference of metabolic profiles between groups. Dark blue: NT group, green: ICS group. (C) Heatmap of some differential metabolites between NT and HC groups. (D) Heatmap of some differential metabolites between ICS group and NT groups. (E,F) Correlations between the most significant differential metabolites between NT and HC groups (E), and between ICS group and NT groups (F). Only these coefficients of *p*‐value < .05 were shown. (G,H) The pathway analysis by MetaboAnalyst on differential metabolites between NT group and HC group (G), between ICS group and NT group (H). Each circle is a representative of a biological pathway, and the size of the circle is enumerated based on the importance

Fungi, bacteria, and metabolites coexist in the gut and modulate the immune system; their interactions may influence human health.^7^ We previously profiled the gut bacteriome in asthma,^4^ we selected the same subsets from bacterial, fungal, and metabolic analysis to establish cross‐domain associations and bacterial–fungal, bacterial–metabolite, and fungal–metabolite co‐occurrence networks. We detected more connections within bacterial and fungal species, and between bacteria and fungi in the NT group than in HC, as evidenced by the former's higher density (.16 vs..05) and number of edges (1010 vs. 587). The figures were lower in the ICS group than that in NT group (.04 vs. .16 and 362 vs. 1010, respectively; Fig. S5–S7). Figure 3 showed that HC had a greater density of bacterial‐metabolite connections than NT and ICS groups. HC and NT group exhibited distinct fungal–metabolite connections, whereas the networks in ICS group were markedly low (Fig. 4), suggesting complex relationships among bacteria, fungi, and metabolites. Moreover, the ICS treatment altered bacterial–metabolite and fungal–metabolite connections.

**FIGURE 3:**
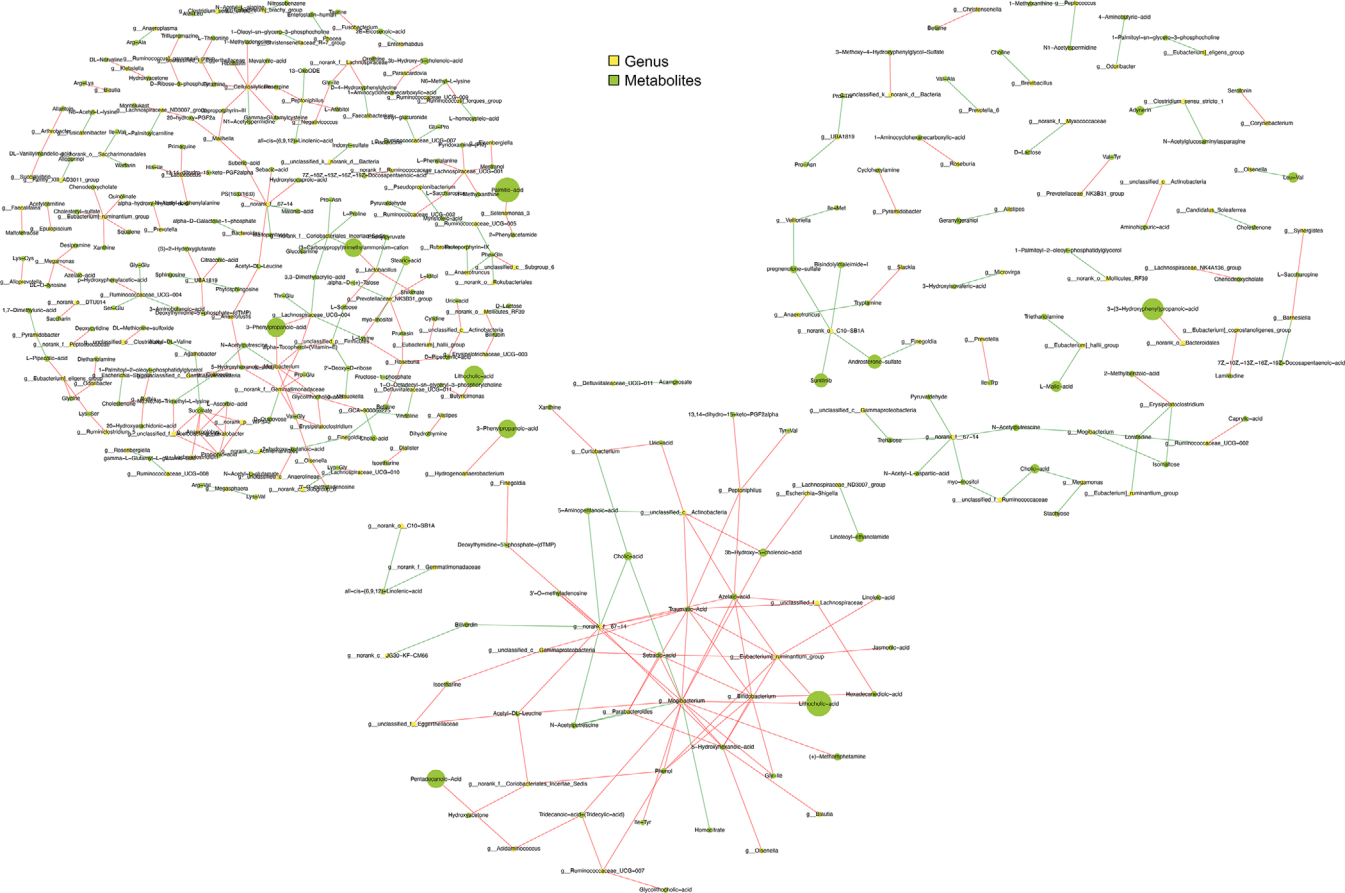
Network analysis of bacteria‐metabolites in fecal samples of healthy control (HC), NT and inhaled corticosteroid (ICS) groups. Network of bacteria–metabolites were performed with Spearman correlation. Each circle (node) represents a microbial genus or metabolites, its size represents the relative abundance. The edge colour indicates the magnitude of the distance correlation; green indicates negative correlation and red indicates positive correlation (determined using spearman test). Upper left: HC group, upper right: NT group, middle: ICS group. Only strong (Spearman *r* > .7) and significant (*p* < .01) correlations are displayed

**FIGURE 4:**
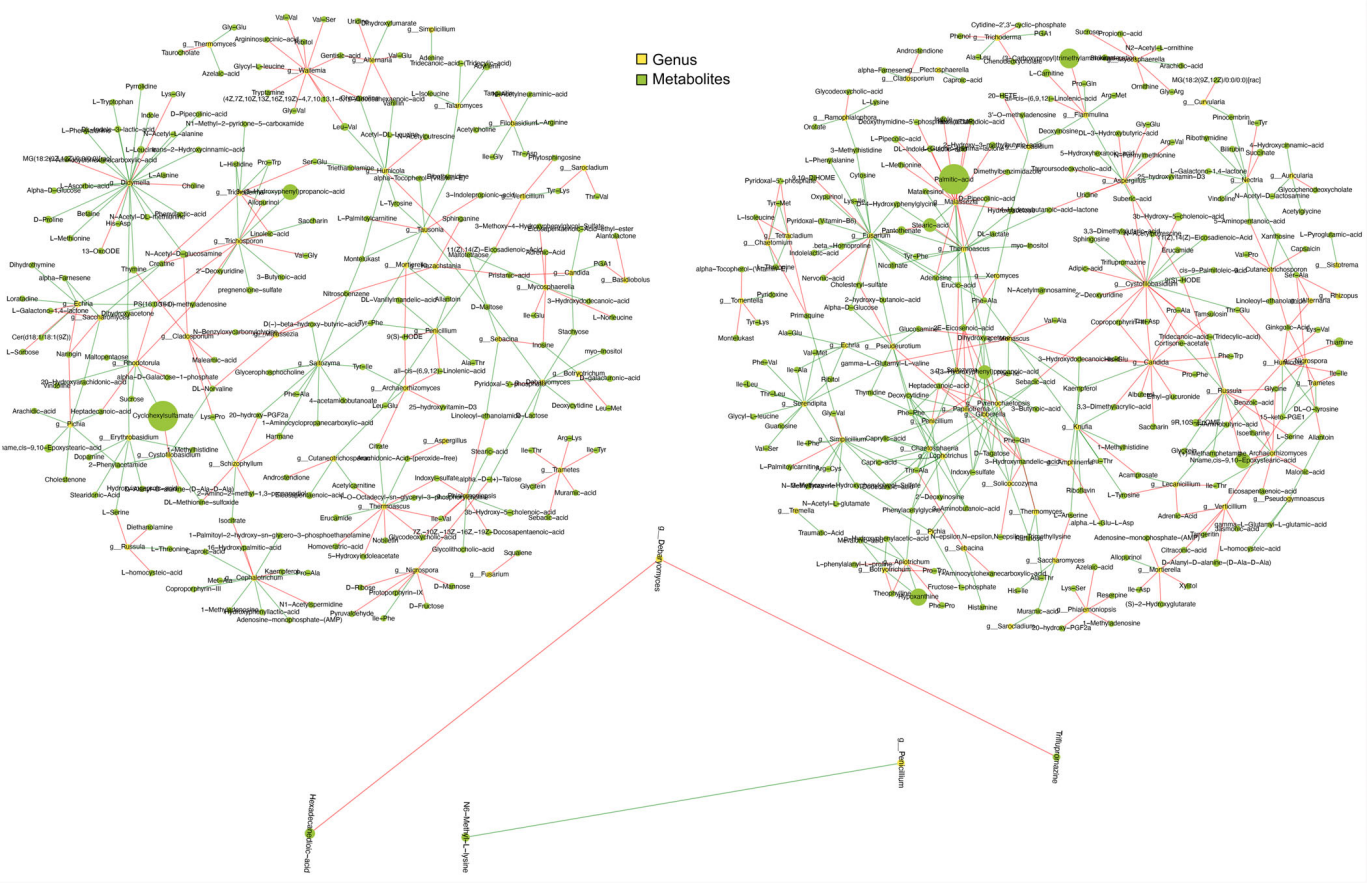
Network analysis of fungi‐metabolites in fecal samples of healthy control (HC), NT and inhaled corticosteroid (ICS) groups. Network of fungi–metabolites were performed with Spearman correlation. Each circle (node) represents a microbial genus or metabolites, its size represents the relative abundance. The edge colour indicates the magnitude of the distance correlation; green indicates negative correlation and red indicates positive correlation (determined using spearman test). Upper left: HC group, upper right: NT group, middle: ICS group. Only strong (Spearman *r* > .68) and significant (*p* < .01) correlations are displayed

Our study was limited by its relatively small‐sample size, a lack of corrections for multiple testing and racially diverse participants, and an independent replication cohort. Because this was not a randomized ICS trial and non‐independent variables of asthma were included in correlation analyses, confounders may account for the observed associations between the mycobiome and ICS or clinical indices (e.g., asthma severity). Therefore, these exploratory findings require validation through large‐scale studies.

In conclusion, asthma patients harboured altered gut fungal compositions, metabolic profiles, and associated metabolic pathways. ICS altered the mycobiome community and fungal/bacterial–metabolite connections. Our findings further our understanding of the gut mycobiome, metabolome, and fungal/bacterial–metabolite associations that may be critical in asthma pathogenesis and may therefore be therapeutic targets.

## CONFLICT OF INTEREST

The authors declare that there is no conflict of interest that could be perceived as prejudicing the impartiality of the research reported.

## FUNDING

the National Natural Science Foundation of China, Grant Numbers: 82170023 and 81970020; Shanghai Sailing Program, Grant Number: 20YF1428300; Shanghai Municipal Health Commission, Grant Number: 2019SY006; Shanghai Key Laboratory of Emergency Prevention, Diagnosis and Treatment of Respiratory Infectious Diseases, Grant Number: 20dz2261100 ; Shanghai Municipal Key Clinical Specialty, Grant Number: shslczdzk02202; Cultivation Project of Shanghai Major Infectious Disease Research Base, Grant Number: 20dz2210500; Shanghai Key Discipline for Respiratory Diseases, Grant Number: 2017ZZ02014; Shanghai Shenkang Hospital Development Center Clinical Science and Technology Innovation Project, Grant Number: SHDC12018102

## Supporting information

Supplementary Materials

Supplementary Figures and Tables
