# Supplementary material for: Disentangling the potential roles of the human gut mycobiome and metabolites in asthma
Source: Clin Transl Med. 2022 Aug 28;12(8):e1012. doi: 10.1002/ctm2.1012 (PMC9420422; doi:10.1002/ctm2.1012)
Supplement: Supplementary file 1 — Supplementary Materials [file CTM2-12-e1012-s002.docx]

**Supplementary Materials**

**Supplementary methods**

**Participants**

All of the asthma patients in their chronic phase were recruited at respiratory department of Ruijin hospital from 2018 to 2020. Patients receiving inhaled corticosteroids (ICS) were recruited from the list of asthma patients that we reviewed. Patients without ICS treatment were diagnosed according to the Global Initiative for Asthma (GINA) Guidelines.

For the microbiome study, 21 healthy participants (HC) and 38 asthma patients were enrolled. All the patients can be grouped into patients without ICS treatment (NT group, n = 12) and patients receiving inhaled corticosteroids (ICS group, n = 26). Patients without ICS treatment in NT group received irregular use of albuterol. All the asthma patients can also be categorized into mild-to-moderate [60% < forced expiratory volume in 1 second percentage predicted (FEV1%pre) <80%, n = 28) and severe patients (FEV1%pre <60%, n = 10) according to GINA guidelines. For some analyses, all asthma patients were classified into three age groups: 18-34 years (n=15), 35-59 years (n=13) and 60-81 years (n=10), or classified by genders (female, n = 15; male, n = 23). For metabolic study, 24 healthy participants were enrolled, 54 asthma patients were recruited and classified into patients not treated with ICS (n = 23) and patients receiving ICS (n = 31).

Patients with the following conditions were excluded from the study: active infections with bacteria, fungi or virus, smoking history, cardiovascular disease, autoimmune diseases, diabetes, intestinal diseases, cancer, other severe comorbidities, taking probiotics or antibiotics within one month prior to recruitment. Healthy participants had normal lung function and were free of smoking history, chronic diseases, acute infection, any respiratory symptoms or taking probiotics or antibiotics within one month prior to recruitment.

The demographical information, including age, sex, body mass index (BMI), history of rhinosinusitis, ICS dose were recorded. All participants completed Asthma Control Questionnaires (ACQ7 score) and lung function test, then ACQ7 score, FEV1% pre, FEV1/forced vital capacity percentage (FEV1/FVC) were recorded. In addition, sputum was induced by hypertonic saline nebulization and collected for cell differential counts, then the percentages sputum eosinophils (%) and neutrophils (%) were recorded.

A written informed consent was signed by every participant, this study was approved by Ethics Committee of Ruijin hospital, Shanghai Jiao Tong University, School of Medicine.

**Microbiome profiling**

**Sample collection and DNA extraction**

Fresh fecal samples were collected in the morning, packed into sterile boxes, and immediately frozen at -80°C for further ITS and metabolome sequencing. DNA extractions were performed using Omega Mag-bind soil DNA kit or QIAamp Powerful Fecal DNA Kit according to the manufacturer's protocol.

DNA was quantified using a NanoDrop ND-2000 spectrophotometer (Thermo Electron Corp.), and the integrity and size were assessed using 1.0% agarose gel electrophoresis on gels containing 0.5 mg/ml of ethidium bromide. Finally, DNA was stored at -20°C.

**Polymerase chain reaction (PCR) analysis and fungal internal transcribed spacer (ITS)/** **16S rRNA sequencing**

The fecal DNA isolated from the clinical samples were amplified using the PCR procedure. For amplification of the ITS genes, 25μL-reaction system containing 5×reaction buffer 5μL, 5×GC buffer 5μL, dNTP（2.5mM） 2μL, Forward primer（10μM）1μL, Reverse primer（10uM）1μL, DNA Template 2μL, ddH2O 8.75μL, Q5 High-Fidelity DNA Polymerase 0.25μL. The reaction was held at 98°C for 2 minutes, followed by 25-30 cycles of 98°C for 15 seconds, 55°C for 30 seconds, 72°C for 30 seconds, and an elongation at 72°C for 5 minutes in an ABI thermocycler. Forward primer: 5’- GGAAGTAAAAGTCGTAACAAGG -3’; reverse primer: 5’- GCTGCGTTCTTCATCGATGC-3’. For 16S rRNA gene sequencing, DNA was subjected to amplification of PCR using primers directed at hypervariable region 3-4 (V3-V4) of the 16S rRNA gene (forward primer: 5’- ACTCCTACGGGAGGCAGCAG -3’and reverse primer: 5’- GGACTACHVGGGTWTCTAAT-3’). The PCR products were purified and quantified. The purified amplicons were pooled in equimolar concentrations and used for paired-end sequencing.

The sequence data analysis was mainly processed using Quantitative Insights into Microbial Ecology (QIIME) software. The paired reads were demultiplexed based on the unique barcodes and then merged, filtered and dereplicated. All the unique sequences were then clustered at 98% (via cluster_size) followed by chimera removing (via uchime_denovo). The non-chimera sequences were re-clustered at 97% to generate operational taxonomic unit (OTU) representative sequences and OTU table. Taxonomy was assigned using the classify-sklearn naïve Bayes taxonomy classifier (https://github.com/QIIME2/q2-feature-classifier) in feature-classifier plugin against the UNITE databases or SILVA databases.

**Mycobiome and bacteriome analysis**

α diversity was assessed by calculating microbial coverage, richness (observed species index), evenness (Pielou_e index) and diversity (Shannon index). β diversity was estimated by calculating the Bray-Curtis and visualized using principal coordinate analysis (PCoA). Adonis testing was applied to figure out whether the microbiome composition was significantly different between different groups. Linear discriminant analysis effect size (LEfSe) was performed to identify microbiome biomarkers (discriminately enriched microbiome) in the fecal samples of the asthma patients and healthy controls at various taxonomic ranks. Linear discriminant analysis (LDA) score of 2 were defined as significantly abundant.

**Metabolome profiling**

**Metabolite extraction**

To extract the metabolites, fecal sample was thawed on ice. Precooled mix (methanol/ acetonitrile/ water, 2:2:1, v/v) was added to the sample. After homogenization, the mixture was incubated at -20 °C for 10 min and then centrifuged at 14,000 g at 4 °C for 20 min. The supernatant was dried in a vacuum evaporator. Before liquid chromatography electrospray ionization tandem mass spectrometry (LC-ESI-MS/MS) analysis, samples were redissolved by 100 μL of acetonitrile/ water (1:1, v/v) followed by centrifugation at 14,000 g at 4 °C for 15 min. The supernatant was used for the detection of metabolites.

**LC-ESI-MS/MS analysis**

For LC-ESI-MS/MS analysis, ultra-high-performance liquid chromatography (UHPLC) separation was carried out using an Agilent 1290 Infinity LC system. Briefly, each 2 μL aliquot of sample was injected at a flow rate of 0.5 mL/min, the column temperature was set at 25 °C. Mobile Phase A was water with 25 mM ammonium acetate, 25 mM aqueous ammonia and Mobile Phase B was 100% acetonitrile. The following gradient was established: 0-0.5 min, 98% B; 0.5-7 min, linearly decreased from 95% B to 65% B; 7-8 min, linearly increased from 65% B to 40% B; 8-9 min, 40% B; 9-9.1 min, linearly increased from 40% B to 95% B; 9.1-12 min, 95% B. The QC samples were inserted into the sample queue to monitor and evaluate the stability of the system and the reliability of experimental data.

The samples were separated by UHPLC and subjected to TOF 6600 mass spectrometer (AB SCIEX). Electrospray ionization (ESI) positive ion and negative ion modes were used for detection. The ESI source conditions were established: ion source gas 1 (Gas1): 60, ion source gas 2 (Gas2): 60, curtain gas (CUR): 30, source temperature: 600°C, ion spray voltage floating (ISVF) at ± 5500 V. The mass range was 60 to 1000 m/z with an accumulation time of 0.20 s per spectra in time-of-flight (TOF) MS scanning; the product ion scan was performed for a mass range of 25-1000 m/z with an accumulation time of 0.05 s per spectra. The high-sensitivity mode of information-dependent acquisition (IDa) in both positive and negative modes (±60 V) was used for MS/MS. The collision energy was normalized to set at 35 ± 15 eV, exclude isotopes within 4 Da, candidate ions to monitor per cycle were set at 10.

**Data processing**

Raw data were converted to a common format (MzXML) by the ProteoWizard MSConvert. The XCMS software was used for peak identification, peak grouping. Annotation of isotopes and adducts were conducted by CAMERA (Collection of Algorithms of Metabolite profile Annotation). In the extracted ion features, only the variables having more than 50% of the nonzero measurement values in at least one group were kept. Compound identification of metabolites was performed by comparing of accuracy m/z value (<25 ppm), and MS/MS spectra with an in-house database established with available authentic standards.

After normalization, the processed data were further analyzed in R package. Orthogonal partial least-squares discriminant analysis (OPLS-DA) were performed to screen the difference of metabolites. The variable importance in the projection (VIP) value of each variable in the OPLS-DA model was calculated to indicate its contribution to the classification. Metabolites with the VIP value >1 was further assessed by Student’s t-test at univariate level to measure the significance of each metabolite, with p < 0.05 considered to be statistically significant.

**Statistical analysis**

Statistical analysis was performed in QIIME, R statistical environment and PRISM software. All data were presented as mean ± SD if not specified. For the clinical characteristics, if continuous variables were normally distributed, data were presented as mean ± SD, student t test or welch t test was used for comparisons, otherwise, data were presented as median (IQR) and a non-parametric test (Wilcoxon signed-rank test) for comparisons was used. Categorical variables in clinical characteristics were presented as n (%) and Chi-Square Test was used for comparisons. Statistical significances of mycobiome were evaluated by Kruskal-Wallis test (with dunn’s post-test) and a p < 0.05 was set as a significance. Fungal-bacterial co-occurrence networks were calculated using SparCC (Sparse Correlations for Compositional data) algorithm [1, 2] (r > 0.6, p < 0.05). Network of bacteria-metabolites (r > 0.7, p < 0.01) and fungi-metabolites (r > 0.68, p < 0.01) were performed with Spearman correlation.

Reference

1. Friedman J, Alm EJ: **Inferring correlation networks from genomic survey data.** *PLoS Comput Biol* 2012, **8:**e1002687.

2. Segal LN, Clemente JC, Tsay JC, Koralov SB, Keller BC, Wu BG, Li Y, Shen N, Ghedin E, Morris A, et al: **Enrichment of the lung microbiome with oral taxa is associated with lung inflammation of a Th17 phenotype.** *Nat Microbiol* 2016, **1:**16031.

**Supplementary results**

**Gut mycobiome in asthma patients with different age and gender**

We investigated the gut mycobiome in asthma patients with different age and gender. No significant difference was observed in fungal coverage, richness, evenness and diversity among asthma patients with different ages (18-34 years, 35-59 years, 60-81 years) (Fig S3A-3D), while significant compositional difference was observed among the three groups in PCoA analysis (Adonis test p=0.027) (Fig S3E, TableS2). No significant differences were observed in α diversity (Fig S4A-4D) and community composition between female and male patients (Fig S4E, Table S2).

**Networks between bacteria and metabolites, or between fungi and metabolites**

Network analysis was conducted to decipher the fungi-metabolites and bacteria-metabolites associations. We identified greater density of connections between nodes in HC group than NT and ICS groups (Fig 3). Firstly, HC group showed many correlations between metabolites and genera Cellulosilyticum, Lachnospiraceae_UCG, norank_f_67-14, Eubacterium_ruminantium_group, Prevotellaceae_NK3B31_group, Mogibacteium, which were reported not to be the core genera correlated with metabolites in NT group except for genus norank_f_67-14 (Fig 3). Genus norank_f_67-14 showed negative connections with Pyruvaldehyde, Trehalose, N-Acetyputrescine, N-Acetyl-L-aspartic_acid and myo-inositol in NT group, which is different from the positive connections with other metabolites in HC group (Fig 3). Globally speaking, more negative bacteria-metabolites associations were detected in NT group. However, bacteria and metabolites showed more positive associations in ICS group, in which the connections from genera Mogibacteium, Bifidobacterium and norank_f_67-14 to metabolites increased compared with NT group (Fig 3), indicating that ICS treatment could reverse bacteria-metabolites connections induced by asthma to some degree.

In fungi-metabolites network, our results indicated that fungi such as genera Didymella, Trichosporon, Wallemia, Trichosporon, Thermoascus, Nigrospora, Sahizophyllum, Cephalotrichum, Penicillium, were some of the main players in the network of HC group (Fig 4). In NT group, we found many connections between metabolites and Malassezia, Fusarium, Aspergillus, Thermoascus, Cystofilobasidium, Candida, Russula, Serendipita, Simplicillium (Fig 4). Strikingly, networks in ICS group were dramatically decreased (Fig 4). The data showed connections from genus Penicillium to N6-Methyl-L-lysine, connections from genus Debaryomyces to Triflupromazine and Hexadecanedioic-acid.

Taken together, these results suggest a complex relationship between bacteria, fungi and metabolites, ICS treatment altered connections of bacteria-metabolites and fungi-metabolites.
