# Supplementary material for: Disentangling the potential roles of the human gut mycobiome and metabolites in asthma
Source: Clin Transl Med. 2022 Aug 28;12(8):e1012. doi: 10.1002/ctm2.1012 (PMC9420422; doi:10.1002/ctm2.1012)
Supplement: Supplementary file 2 — Supplementary Figures and Tables [file CTM2-12-e1012-s001.docx]

**Figure S1. Taxonomic distribution of enteric fungi among the three group at the class and family levels.
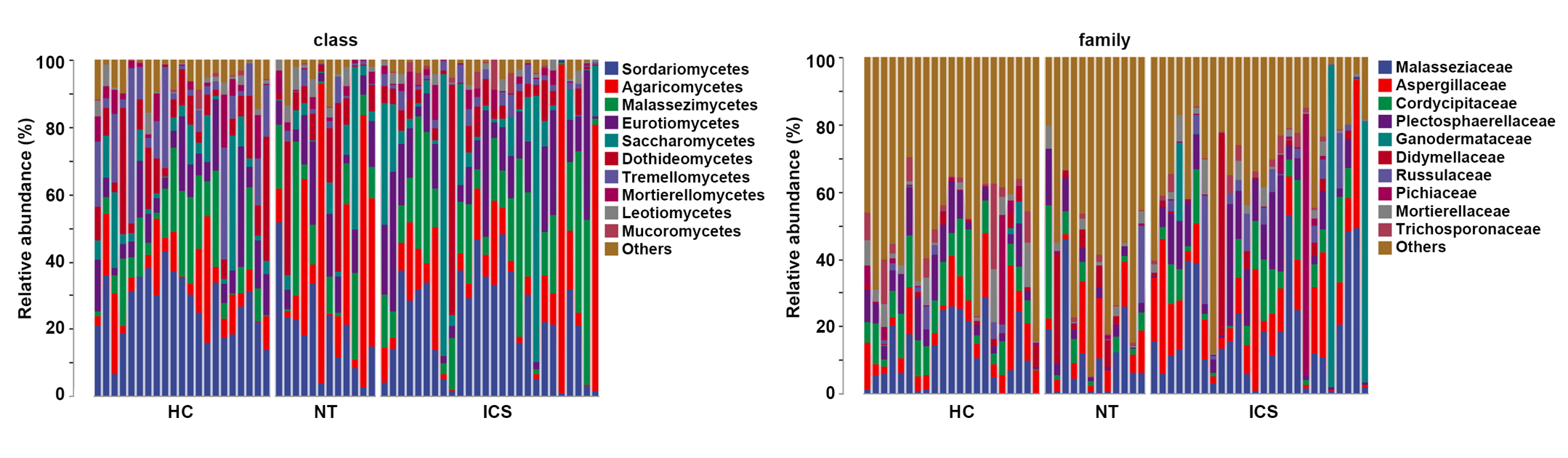
**

**Figure S2. α and β diversity of gut mycobiome among HC, mild-to-moderate and severe groups.**

A-D. Comparison of α diversity including species coverage (A), observed species (B), Pielou_e (C), Shannon (D) metrics among HC (n = 21), mild-to-moderate (n = 28) and severe groups (n = 10). Kruskal-Wallis test with dunn’s post-test. *p<0.05. **p<0.01. E. Principal coordinate analysis (PCoA) of Bray-Curtis distance matrix analysis.


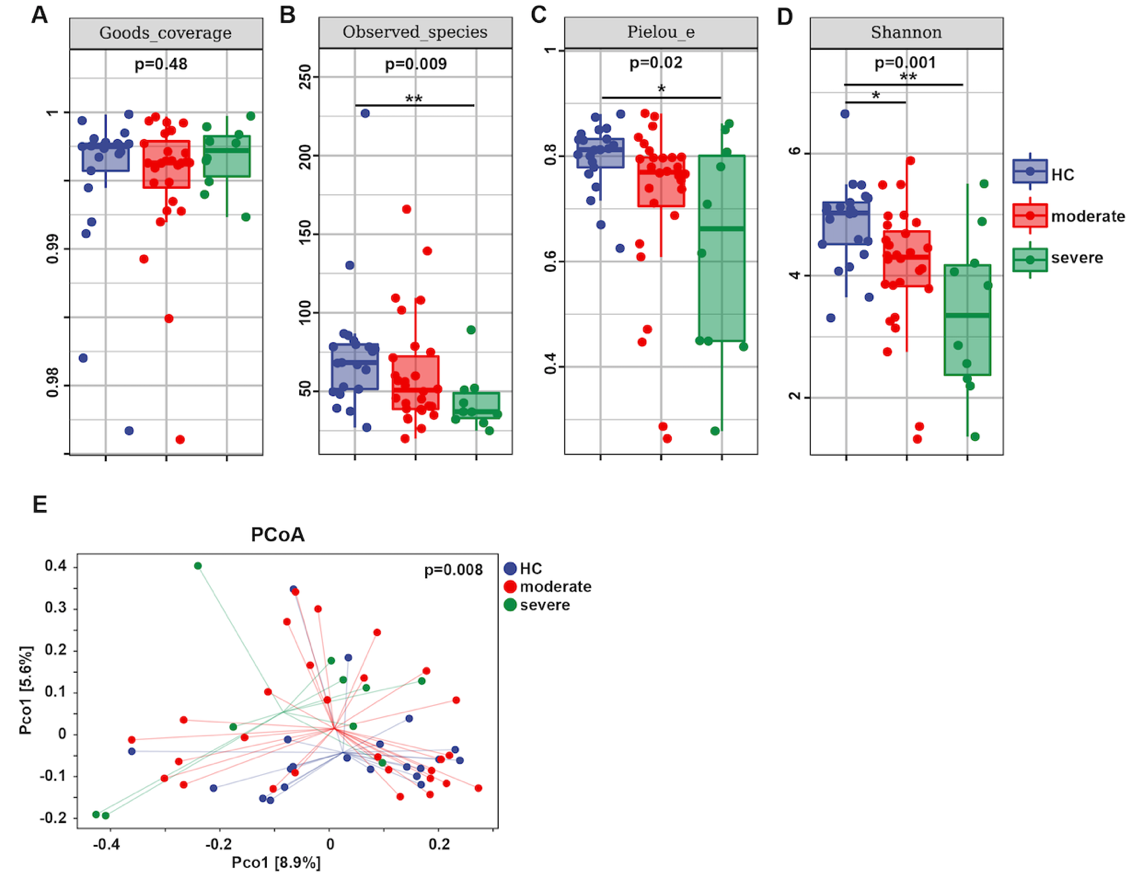


**Figure S3. Comparison of α (A) and β diversity (B) in asthma patients with different age.**

A-D. Comparison of α diversity including species coverage (A), observed species (B), Pielou_e (C), Shannon (D) metrics among patients with different ages (18-34 years, n = 15; 35-59 years, n = 13); 60-81 years, n = 10). Kruskal-Wallis test with dunn’s post-test. E. Principal coordinate analysis (PCoA) of Bray-Curtis distance matrix analysis.

**
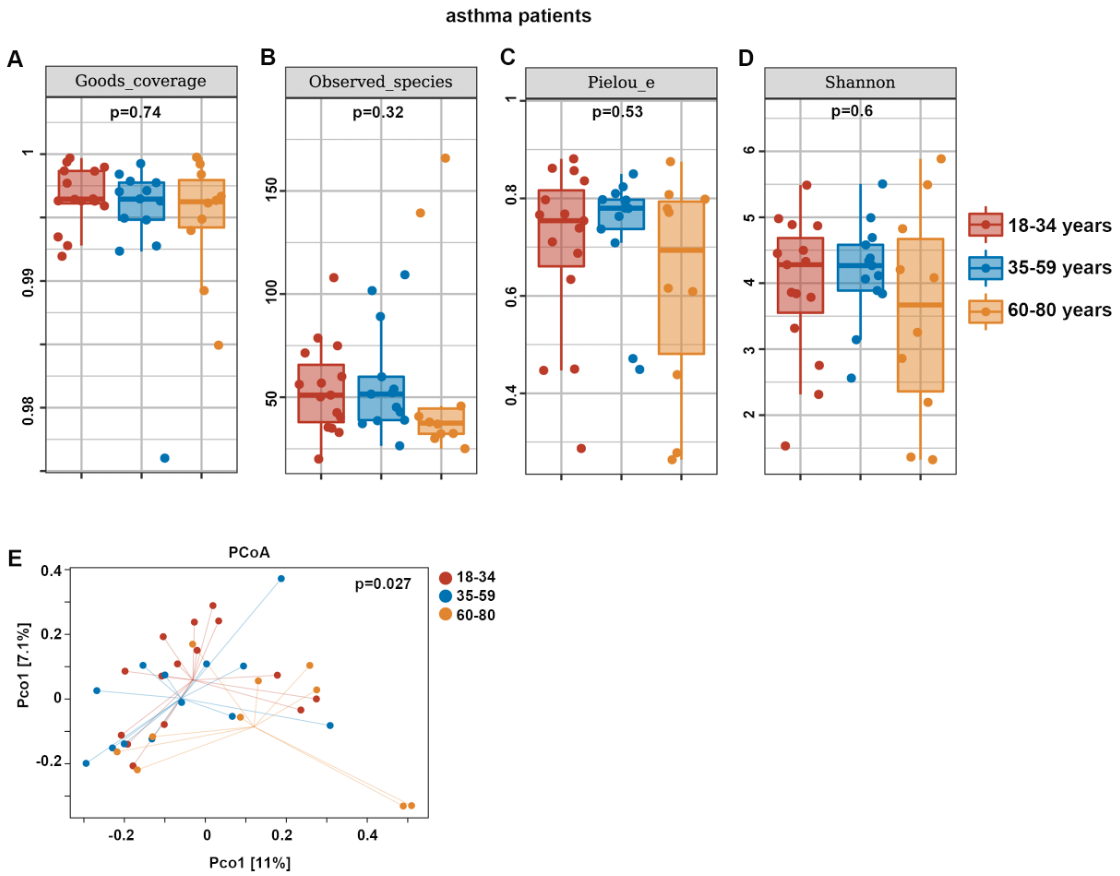
**

**Figure S4. Comparison of α and β diversity in female and male patients.**

A-D. Comparison of α diversity including species coverage (A), observed species (B), Pielou_e (C), Shannon (D) metrics among between female (n = 15) and male (n = 23) patients. Kruskal-Wallis test with dunn’s post-test. E. PCoA of Bray-Curtis distance matrix analysis.

**
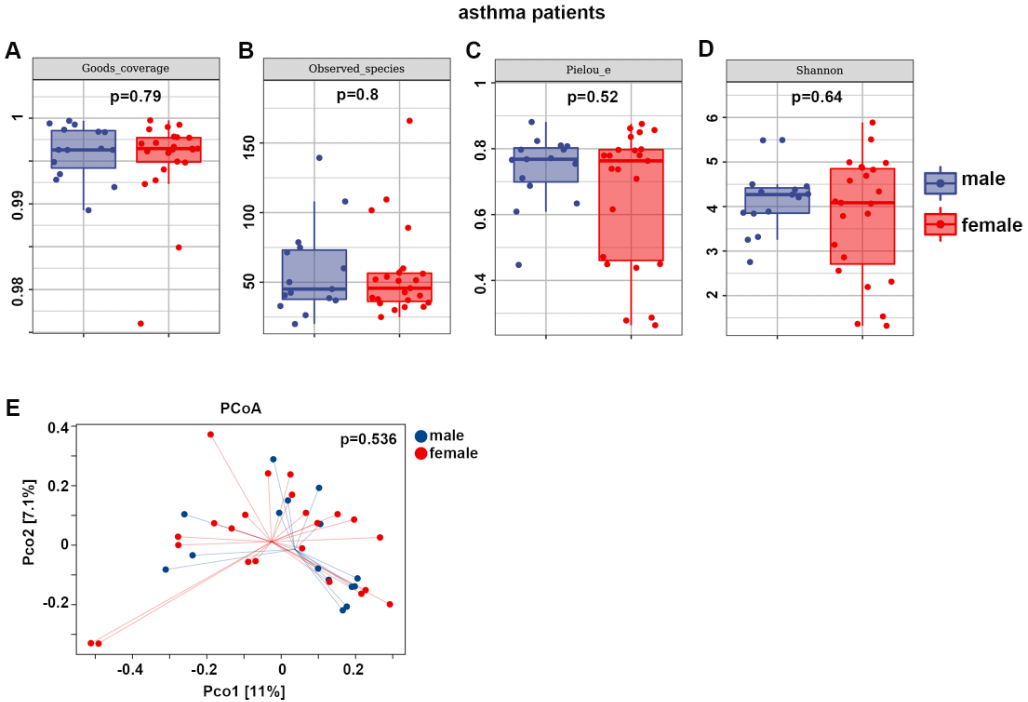
**

**Figure S5. Fungal-bacterial co-occurrence networks in fecal samples of HC group.**

Networks were calculated using SparCC (Sparse Correlations for Compositional data) algorithm. Each circle (node) represents a bacterial or fungal genus, the node colour represents the phylum it belongs to. The edge colour indicates the magnitude of the distance correlation; green indicates positive correlation and red indicates negative correlation. Only strong (r>0.6) and significant (p<0.05) correlations are displayed.

**
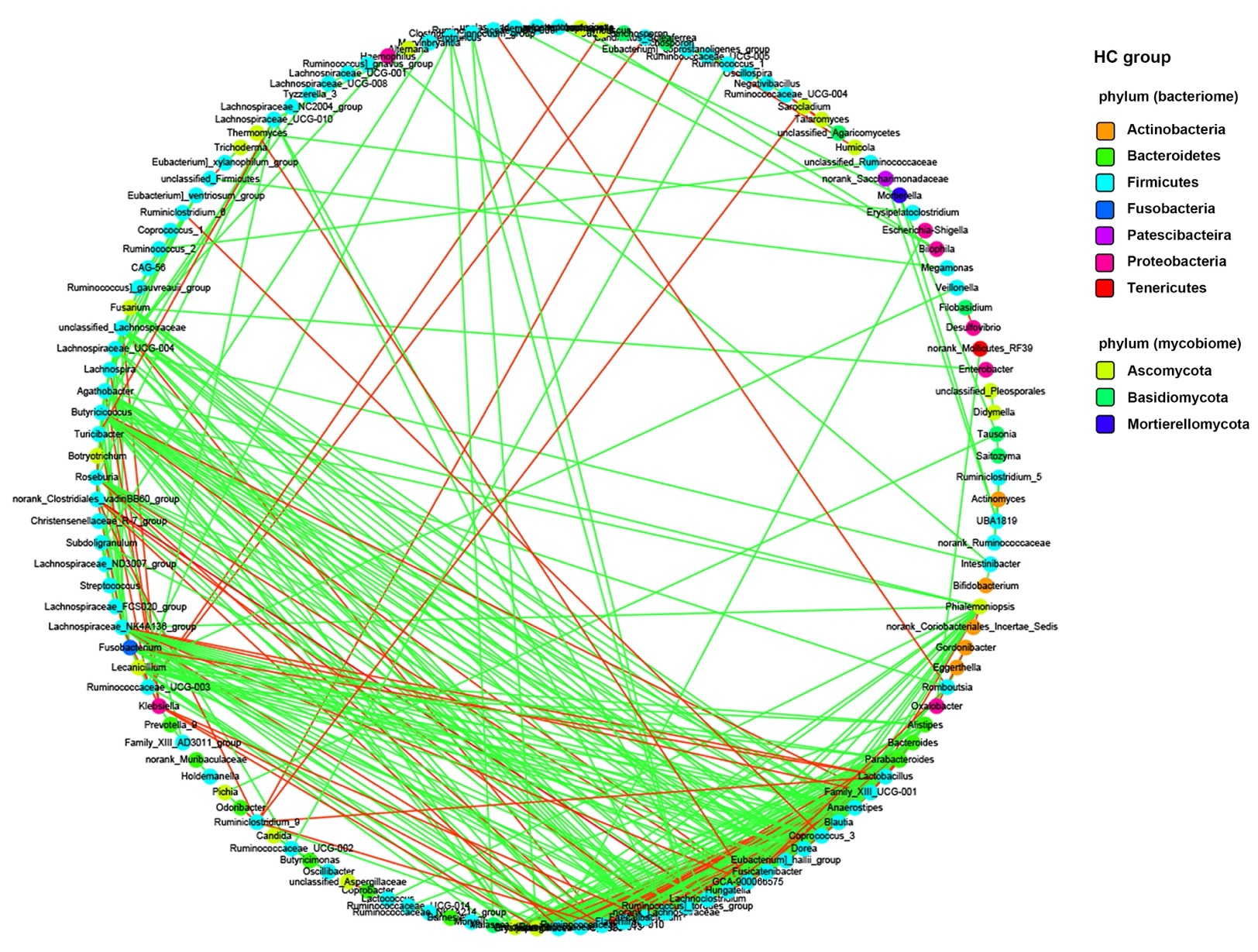
**

**Figure S6. Fungal-bacterial co-occurrence networks in fecal samples of NT group.**

Networks were calculated using SparCC (Sparse Correlations for Compositional data) algorithm. Each circle (node) represents a bacterial or fungal genus, the node colour represents the phylum it belongs to. The edge colour indicates the magnitude of the distance correlation; green indicates positive correlation and red indicates negative correlation. Only strong (r>0.6) and significant (p<0.05) correlations are displayed.


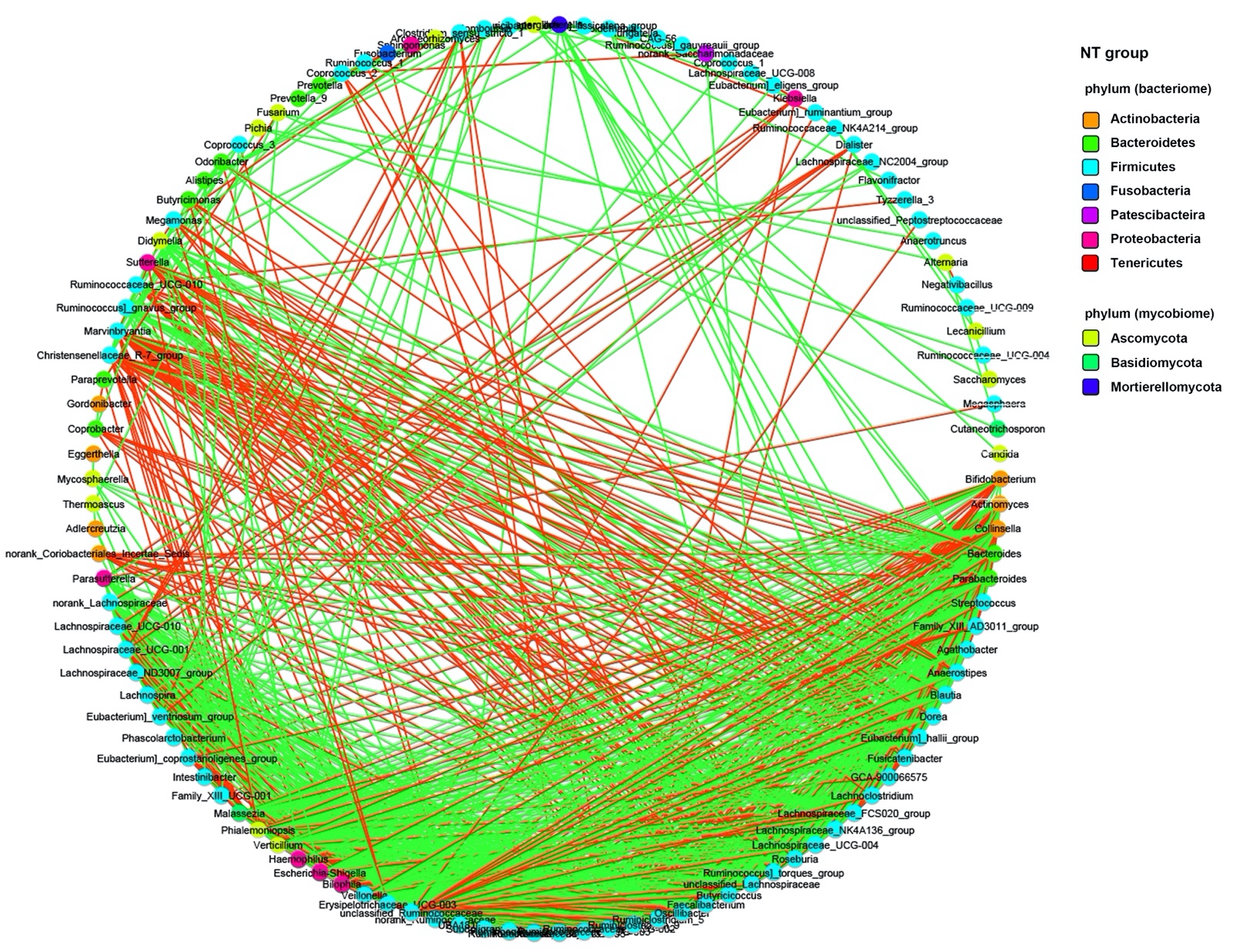


**Figure S7. Fungal-bacterial co-occurrence networks in fecal samples of ICS group.**

Networks were calculated using SparCC (Sparse Correlations for Compositional data) algorithm. Each circle (node) represents a bacterial or fungal genus, the node colour represents the phylum it belongs to. The edge colour indicates the magnitude of the distance correlation; green indicates positive correlation and red indicates negative correlation. Only strong (r>0.6) and significant (p<0.05) correlations are displayed.

**
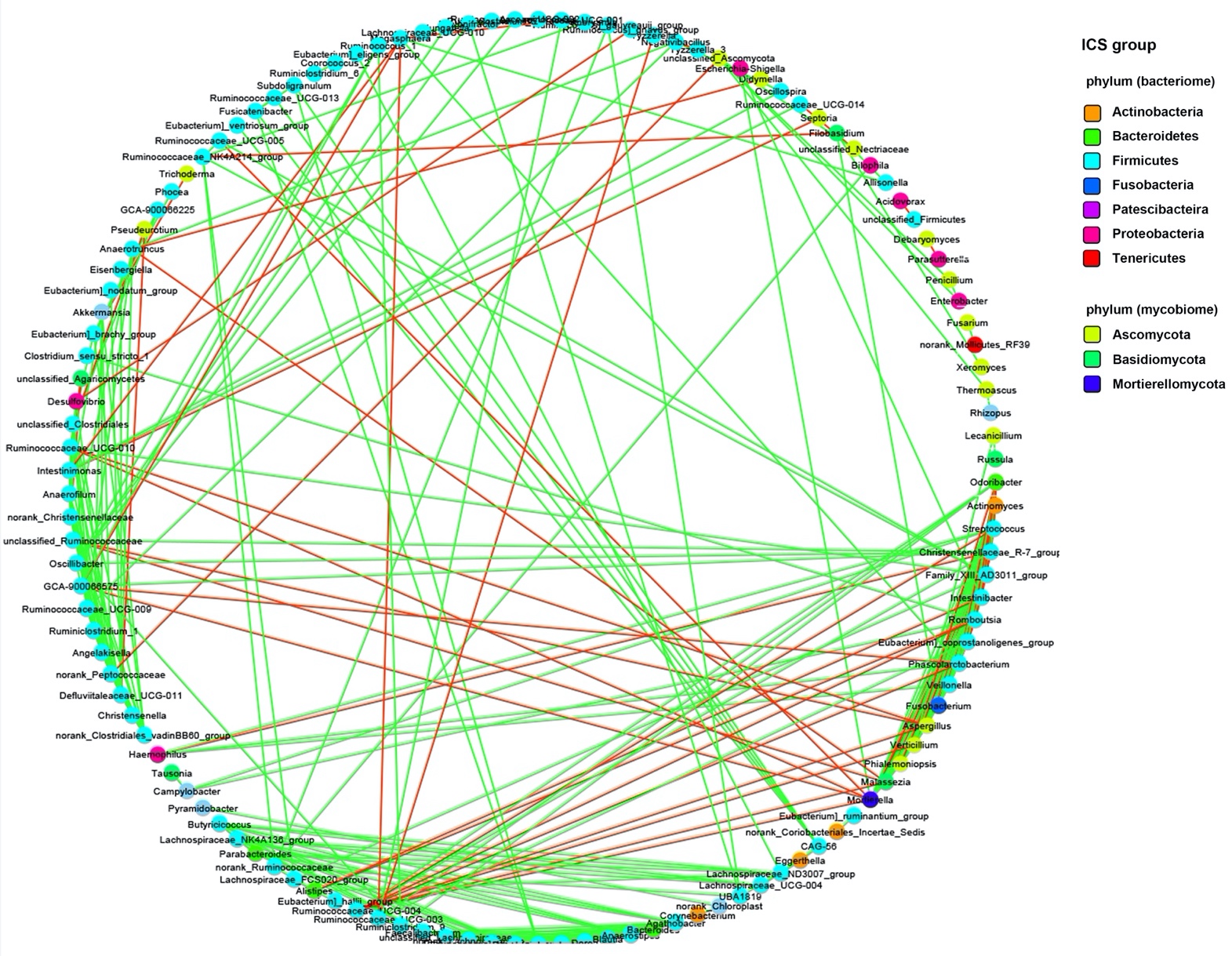
**

**Table S1. Demographic and clinical characteristics of study subjects in mycobiome analysis (n=59)**

|  | HC (n=21) | NT (n=12) | ICS (n=26) | P value  NT vs HC | P value  ICS vs HC | P value  ICS vs NT |
| --- | --- | --- | --- | --- | --- | --- |
| Age, years, mean ± SD | 40.0±9.2 | 42.1±17.0 | 45.7±15.1 | 0.93 | >0.99 | 0.52 |
| Male, no. (%) | 11 (52.4) | 3 (25) | 12 (46.2) | 0.16 | 0.77 | 0.29 |
| BMI, kg/m2, mean ± SD | 23.1±2.7 | 23.7±4.1 | 22.9±2.9 | 0.40 | 0.43 | 0.12 |
| Duration of asthma, years, median (IQR) | - | 0.06 (0.008, 0.91) | 2 (0.73, 11.5) | - | - | 0.001 |
| Diet: Omnivore, no. (%) | 21 (100) | 12 (100) | 26 (100) | - | - | - |
| Allergic rhinitis, no. (%) | 0 (0) | 9 (75.0) | 16 (61.5) | - | - | 0.48 |
| ICS dose^1^, μg.day-1, median (IQR) | - | - | 400 (400, 725) | - | - | - |
| FEV1 (% predicted), mean ± SD | 96.4 ± 9.3 | 77.0±22.4 | 80.6 ±19.6 | 0.02 | 0.001 | 0.63 |
| FEV1/FVC (%), mean ± SD | 81.7 ± 6.4 | 73.2±10.3 | 73.4 ± 13.5 | 0.01 | 0.01 | 0.92 |
| ACQ7 score, mean ± SD | - | 1.2 ± 0.8 | 0.8 ± 0.5 | - | - | 0.15 |
| Sputum eosinophils (%), median (IQR) | 1 (0, 2) | 1.5 (0.87, 2.62) | 1 (0.12, 2.37) | 0.08 | 0.36 | 0.26 |
| Sputum neutrophils (%), median (IQR) | 24.5 (6, 46.75) | 15.75 (3.87, 38.12) | 10 (7.5, 33) | 0.48 | 0.23 | 0.89 |

^1^ ICS dose was expressed as beclomethasone propionate equivalent dose.

If continuous variables were normally distributed, data were presented as mean ± SD, student t test or welch t test was used for comparisons, otherwise, data were presented as median (IQR) and a non-parametric test (Wilcoxon signed-rank test) for comparisons was used. Categorical variables were presented as n (%) and Chi-square Test was used for comparisons.

**Table S2. Adonis testing based on Bray-Curtis distance.**

| Groups | Df | SS | MS | F | R2 | P |
| --- | --- | --- | --- | --- | --- | --- |
| HC/NT/ICS | 2 | 0.959653 | 0.479826 | 1.285909 | 0.043909 | 0.026 |
| HC/ mild-to-moderate/ Severe | 2 | 1.037612 | 0.518806 | 1.395579 | 0.047476 | 0.008 |
| Age groups | 2 | 1.01411 | 0.507055 | 1.341693 | 0.071209 | 0.027 |
| Male/ female | 1 | 0.372076 | 0.372076 | 0.965782 | 0.026126 | 0.536 |

**Table S3. Demographic and clinical characteristics of study subjects in metabolome analysis (n=78)**

|  | HC (n=24) | NT (n=23) | ICS (n=31) | P value  NT vs HC | P value  ICS vs HC | P value  ICS vs NT |
| --- | --- | --- | --- | --- | --- | --- |
| Age, years, median (IQR) | 42 (32.5, 49.25) | 34 (27, 56) | 43 (34, 59) | 0.46 | 0.27 | 0.19 |
| Male, no. (%) | 14 (58.3) | 10 (43.5) | 12 (38.7) | 0.38 | 0.18 | 0.78 |
| BMI, kg/m2, mean ± SD | 23.1±3.3 | 22.8±2.8 | 22.7±2.7 | 0.51 | 0.69 | 0.30 |
| Duration of asthma, years, median (IQR) | - | 0.08 (0.01, 0.5) | 2 (0.79, 11) | - | - | <0.0001 |
| Diet: Omnivore, no. (%) | 24 (100) | 23 (100) | 31 (100) | - | - | - |
| Allergic Rhinitis, no. (%) | 0 (0) | 18 (78.2) | 22 (71.0) | - | - | 0.75 |
| ICS dose^1^, μg.day-1, median (IQR) | - | - | 400 (400, 1000) | - | - | - |
| FEV1 (% predicted), mean ± SD | 95.9±10.1 | 70.5±21.7 | 81.8 ±20.1 | <0.0001 | 0.0017 | 0.05 |
| FEV1/FVC (%), mean ± SD | 82.7 ± 6.9 | 69.7±11.5 | 73.3 ± 13.7 | <0.0001 | 0.002 | 0.31 |
| ACQ7 score, median (IQR) | - | 1.1 (0.8, 2) | 0.7 (0.4, 1.0) | - | - | 0.0042 |
| Sputum eosinophils (%), median (IQR) | 0.5 (0, 1.6) | 2 (1, 3.5) | 1.5 (0.5, 2.2) | 0.0006 | 0.10 | 0.04 |
| Sputum neutrophils (%), median (IQR) | 20.2 (3.6, 46.7) | 12.5 (3.7, 36.2) | 12.5 (8, 32.2) | 0.41 | 0.46 | 0.77 |

^1^ ICS dose was expressed as beclomethasone propionate equivalent dose.

If continuous variables were normally distributed, data were presented as mean ± SD, student t test or welch t test was used for comparisons, otherwise, data were presented as median (IQR) and a non-parametric test (Wilcoxon signed-rank test) for comparisons was used. Categorical variables were presented as n (%) and Chi-square Test was used for comparisons.

**Table S4. Identification results of significant metabolites between NT and HC groups.**

| **ID** | **Adduct** | **Metabolites** | **VIP** | **Fold change**  **(NT vs HC)** | **p-value** | **m/z** | **rt(s)** | **Class** |  |
| --- | --- | --- | --- | --- | --- | --- | --- | --- | --- |
| M162T356_2 | (M+H) + | L-Camitine | 4.52352015 | 1.94552206 | 0.01194241 | 162.1127 | 356.116 | Organonitrogen compounds |  |
| M365T384 | (M+Na) + | Cellobiose | 2.0313592 | 0.37358398 | 0.0180686 | 365.105 | 383.78 | Organooxygen compounds |  |
| M300T88 | (M+H) + | Sphingosine | 4.40627269 | 3.674973 | 0.01846736 | 300.28951 | 87.8985 | Organonitrogen compounds |  |
| M264T167 | (M+H-H2O) + | 3’-O-methyladenosine | 1.05737622 | 0.51102343 | 0.02033551 | 264.10902 | 166.753 | Purine nucleosides |  |
| M269T33 | (M+H-H2O) + | Retinol (Vitamin A) | 1.04394377 | 0.50184882 | 0.0204667 | 269.22617 | 33.4555 | **/** |  |
| M306T36 | (M+H) + | Capsaicin | 1.0644098 | 0.31994105 | 0.0289433 | 306.20741 | 35.745 | Phenols |  |
| M166T375 | (M+H) + | DL-Methionine sulfoixide | 1.04241455 | 0.68079999 | 0.03254671 | 166.05287 | 375.266 | Carboxylic acids and derivatives |  |
| M295T103 | (M+H-H2O) + | Tyr-Met | 1.4563831 | 2.5827164 | 0.0387642 | 295.11532 | 102.626 | **/** |  |
| M145T293 | (M+H) + | N-Acetylcadaverine | 4.12322066 | 0.35244635 | 0.04618321 | 145.13323 | 293.141 | Carboxylic acids and derivatives |  |
| M141T339_2 | (M-H2O-H)- | 2-Oxoadipic acid | 5.48508019 | 0.49654015 | 0.02331747 | 141.0171 | 339.289 | Keto acids and derivatives |  |
| M163T130 | (M-H)- | L-Fucose | 1.03793894 | 0.61672938 | 0.02552633 | 163.0604 | 130.341 | Organooxygen compounds |  |
| M313T42 | (M-H)- | 9,10-DiHOME | 1.96686151 | 0.67264535 | 0.04773093 | 313.23622 | 42.392 | / |  |

**Table S5. Identification results of significant metabolites between ICS and NT groups.**

| **ID** | **Adduct** | **Metabolites** | **VIP** | **Fold change**  **(ICS vs NT)** | **p-value** | **m/z** | **rt(s)** | **Class** |  |
| --- | --- | --- | --- | --- | --- | --- | --- | --- | --- |
| M229T242 | (M+H)+ | Ile-Pro | 1.97250602 | 0.65882474 | 0.03404893 | 229.15492 | 242.229 | **/** |  |
| M132T402 | (M+H)+ | Creatine | 1.16824855 | 2.00721425 | 0.04597732 | 132.0764 | 402.1 | Carboxylic acids and derivatives |  |
| M264T167 | (M+H-H2O)+ | 3’-O-methyladenosine | 1.07821807 | 1.68087448 | 0.04743897 | 264.10902 | 166.753 | Purine nucleosides |  |
| M495T49 | (M-H)- | Enterostatin human | 1.80038292 | 0.67494686 | 0.01899541 | 495.27518 | 48.947 | / |  |
| M178T70 | (M-H)- | Cyclohexylsulfamate | 42.1143457 | 5.14574153 | 0.02181123 | 178.05369 | 69.8755 | **/** |  |
| M932T27 | (2M-H)- | Cholesteryl sulfate | 1.16445852 | 0.65165176 | 0.03269018 | 931.6127 | 26.511 | / |  |
| M411T31_2 | (M-H)- | Bisindolylmaleimide I | 2.12243971 | 0.44211952 | 0.04632153 | 411.18142 | 31.144 | / |  |
| M395T30 | (M-H)- | pregnenolone sulfate | 3.32050002 | 0.51017852 | 0.04657595 | 395.18707 | 29.529 | Steroids and steroid derivatives |  |
| M397T32_2 | (M-H)- | Sunitinib | 6.80028837 | 0.53553969 | 0.04984414 | 397.20356 | 31.7 | / |  |
